# The thalamus and its subregions – a gateway to obsessive-compulsive disorder

**DOI:** 10.1192/j.eurpsy.2022.239

**Published:** 2022-09-01

**Authors:** C. Weeland, C. Vriend, Y. Van Der Werf, C. Huyser, M. Hillegers, H. Tiemeier, T. White, N. De Joode, P. Thompson, D. Stein, O. Van Den Heuvel, S. Kasprzak

**Affiliations:** 1 Amsterdam UMC, Department Of Anatomy And Neurosciences; Department Of Psychiatry, Amsterdam, Netherlands; 2 Academic Center for Child and Adolescent Psychiatry, De Bascule, Amsterdam, Netherlands; 3 Erasmus Medical Center, Department Of Child And Adolescent Psychiatry, Rotterdam, Netherlands; 4 Harvard TH Chan School of Public Health, -, Boston, United States of America; 5 Erasmus MC, Child And Adolescent Psychiatry And Psychology, Rotterdam, Netherlands; 6 eImaging Genetics Center, Stevens Institute for Neuroimaging & Informatics, Keck School of Medicine, University of Southern California, -, Los Angeles, United States of America; 7 University of Cape Town, Dept Of Psychiatry & Neuroscience Institute, Cape Town, South Africa

**Keywords:** OCD, thalamus, Neuroimaging, segmentation

## Abstract

**Introduction:**

Higher thalamic volume has been found in children with obsessive-compulsive disorder (OCD) and children with clinical-level symptoms within the general population (Boedhoe et al. 2017, Weeland et al. 2021a). Functionally distinct thalamic nuclei are an integral part of OCD-relevant brain circuitry.

**Objectives:**

We aimed to study the thalamic nuclei volume in relation to subclinical and clinical OCD across different age ranges. Understanding the role of thalamic nuclei and their associated circuits in pediatric OCD could lead towards treatment strategies specifically targeting these circuits.

**Methods:**

We studied the relationship between thalamic nuclei and obsessive-compulsive symptoms (OCS) in a large sample of school-aged children from the *Generation R Study* (N = 2500) (Weeland et al. 2021b). Using the data from the ENIGMA-OCD working group we conducted mega-analyses to study thalamic subregional volume in OCD across the lifespan in 2,649 OCD patients and 2,774 healthy controls across 29 sites (Weeland et al. 2021c). Thalamic nuclei were grouped into five subregions: anterior, ventral, intralaminar/medial, lateral and pulvinar (Figure 1).

**Figure:**
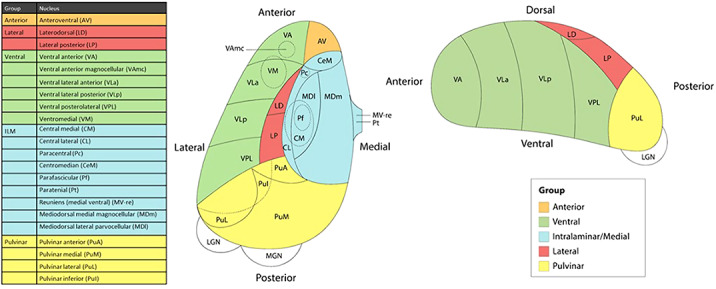

**Results:**

Both children with subclinical and clinical OCD compared with controls show increased volume across multiple thalamic subregions. Adult OCD patients have decreased volume across all subregions (Figure 2), which was mostly driven by medicated and adult-onset patients.

**Figure:**
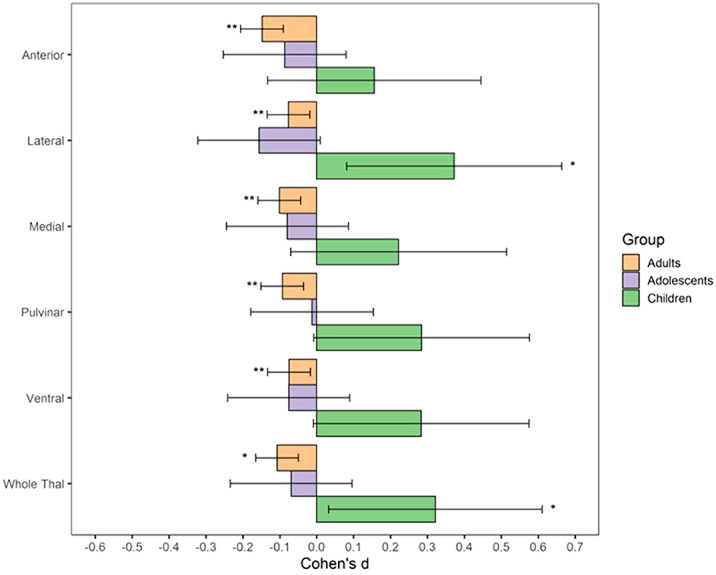

**Conclusions:**

Our results suggests that OCD-related thalamic volume differences are global and not driven by particular subregions and that the direction of effects are driven by both age and medication status.

**Disclosure:**

No significant relationships.

